# Association and Agreement between Nutrition-Focused Physical Examination-Assessed and Bioelectrical Impedance Analysis-Derived Muscle and Fat Status in Patients with Breast Cancer

**DOI:** 10.21315/mjms-02-2026-108

**Published:** 2026-06-30

**Authors:** Seong Ting Chen, Vanessa Shi Ming Wong, Raflis Ruzairee Awang, Krystal Lu Shin Ng, Ting Xuan Wong

**Affiliations:** 1 Division of Nutrition, Dietetics and Food Science, School of Health Sciences, IMU University, Bukit Jalil, 57000 Kuala Lumpur, Malaysia; 2 Department of General Surgery, Hospital Kuala Lumpur, Jalan Pahang, 50586 Kuala Lumpur, Malaysia; 3 Department of Dietetics, National Cancer Society Malaysia, Jalan Raja Muda Abdul Aziz, 50300 Kuala Lumpur, Malaysia

**Keywords:** bioelectrical impedance analysis, breast cancer, fat, muscle, nutrition-focused physical examination

## Abstract

**Background:**

Breast cancer, one of the most common cancers among women in Malaysia, may contribute to the development of sarcopenia through chronic inflammation, which activates muscle protein degradation pathways. Nutrition-focused physical examination (NFPE) has gained increasing attention in clinical settings for body composition assessment, as it enables bedside evaluation without dependence on bioelectrical impedance analysis (BIA), which may be affected by hydration status and limited device availability. This study aimed to assess the association and agreement between NFPE-assessed and BIA-derived muscle and fat status in patients with breast cancer.

**Methods:**

This cross-sectional study included 74 women. NFPE evaluated upper- and lower-body muscle mass and subcutaneous fat stores using inspection and palpation techniques. Body composition was measured using BIA, with participants lying in the supine position as a low-level electrical current was applied. Associations and agreement between muscle and fat status assessed by NFPE and BIA were evaluated using McNemar’s test and Cohen’s kappa analysis, respectively. Statistical significance was set at *P* < 0.05.

**Results:**

NFPE-assessed muscle and fat status were significantly associated with BIA-derived appendicular skeletal muscle index and total body fat, respectively (both *P* < 0.001). Agreement between the two methods was slight by kappa interpretation, with unweighted Cohen’s kappa coefficients of 0.033 for muscle status and 0.011 for fat status.

**Conclusion:**

Although agreement between the two methods was limited, NFPE remains clinically relevant as an accessible and cost-effective first-line screening approach, especially in settings with limited access to BIA.

## Introduction

Breast cancer is the most common malignancy among women globally, and although its incidence is higher in developed countries, it remains a major public health concern in Malaysia. The mortality rate of cancer in Malaysia is elevated because of the high proportion of late-stage diagnoses (1). According to the 2020 Global Cancer Burden and Strategies for Cancer Control report, breast cancer accounts for 32.9% of all cancer cases among Malaysian women (2, 3). Sarcopenia is an important clinical concern in this context and, as defined by the Asian Working Group for Sarcopenia, refers to an age-related decline in muscle mass, strength, and/or physical performance. This condition may be aggravated by the chronic inflammation associated with cancer, leading to muscle degradation and malnutrition (4–6).

Sarcopenia has become a critical concern in cancer patients, as it is associated with higher mortality rates and negatively affects energy balance, muscle function and physical capacity (7, 8). A study of 471 women with stage 1 to 3 breast cancer revealed that 16% had sarcopenia, which served as an independent predictor of reduced survival (9). Furthermore, sarcopenia in cancer patients is linked to increased vulnerability to chemotherapy-related toxicity, frailty and a decline in performance status (5–7). These complications increase the risk of falls, fractures and postoperative complications (5, 10).

Bioelectrical impedance analysis (BIA) is widely recommended for assessing body composition due to its high reliability and accuracy. The BIA analyser is non-invasive and portable, making it well suited for clinical applications (11) and can measure various parameters, including fat mass, fat-free mass, total body water, extracellular water, intracellular water, basal metabolic rate and phase angle (11, 12). BIA operates on the principle of electrical conductivity, in which a low-level alternating current is passed through the body, and the resulting resistance varies according to the current’s frequency (12). Compared with dual-energy X-ray absorptiometry (DEXA), BIA is a less resource-intensive method for evaluating body composition and overall health status (12). It demonstrates a strong correlation with fat mass percentage, making it a valuable tool for estimating body fat. Furthermore, BIA devices can assess segmental body composition, such as lean mass distribution in the arms, trunk and legs (12). Despite its advantages, the cost and limited availability of BIA equipment may restrict its use in local clinical settings (12).

The nutrition-focused physical exam (NFPE) is a comprehensive assessment aimed at identifying the physical signs associated with nutrient deficiencies (13, 14). It involves the evaluation of muscle mass and subcutaneous fat stores; physical characteristics, such as hair, skin, eyes, oral cavity and fingernails; signs of oedema or ascites; and functional status assessed through handgrip strength (13). NFPE plays a critical role in identifying factors that may affect dietary intake and nutritional status (14). It has evolved into a structured clinical tool for assessing physical appearance and body composition and is now widely used to evaluate nutritional status in clinical settings. The primary techniques used in NFPE, inspection and palpation, are performed by a trained dietitian.

In this study involving patients with breast cancer at risk of sarcopenia, particular emphasis was placed on the assessment of muscle and fat status due to the impact of malnutrition on body composition and physical function. The key markers identified included indicators of muscle wasting, such as reduced handgrip strength as a marker of functional status, as well as indicators of fat loss (13). These changes in muscle and fat status, characterised by reductions in bulk and tone, can be detected by palpation (14). NFPE offers a cost-effective and practical method for evaluating muscle and fat stores, assessing functional status, identifying oedema and detecting micronutrient deficiencies (13, 14). NFPE’s affordability and ease of application have contributed to its increased use in clinical settings as a practical surrogate measure for body composition assessment (13).

This study aimed to assess the association and agreement between NFPE-assessed and BIA-derived muscle and fat status in patients with breast cancer. We also aimed to examine the association and agreement between muscle mass, handgrip strength and the presence of sarcopenia.

## Methods

### Study Design and Participants

This cross-sectional study was conducted at Kuala Lumpur Hospital and the National Cancer Society Malaysia from December 2023 to March 2024. Study participants were recruited based on the following inclusion criteria: i) female aged 18 years or older; ii) a primary diagnosis of stage 1 to 3 breast cancer; and iii) willingness to participate in physical examination and BIA. A sample size calculation using Pearson correlation for a moderate correlation (*r* = 0.30) yielded an estimated 67 participants (15); to account for a potential 20% dropout rate, a minimum of 80 participants was targeted.

### Data Collection

#### Personal Data

The participants’ sociodemographic information, including date of birth, ethnicity and monthly household income, was obtained through structured interviews. Medical data, such as the date of initial cancer diagnosis, cancer type and stage, previous and current treatment plans and ongoing medications, were extracted from medical records.

#### Anthropometry and Handgrip Strength

The participants’ weight was measured using a digital weighing scale with an accuracy of 0.1 kg, and their height was measured with a SECA stadiometer, precise to 0.1 cm. Body mass index (BMI) was calculated using the formula weight (kg) / height^2^ (m^2^). Muscle strength was evaluated using the handgrip strength test with a Jamar dynamometer (JAMAR, Roylan, UK). The dominant and nondominant hands were identified, and measurements were taken for each. The participants were seated with elbows flexed at 90° and instructed to perform maximal voluntary isometric contraction using a handgrip dynamometer. The test was conducted twice for each hand, with a one-minute rest interval between the trials to reduce fatigue. The average of these measurements was recorded in kg of force (16).

#### NFPE

NFPE was conducted by dietetics trainees to evaluate: i) muscle wasting; and ii) subcutaneous fat loss in the head and face, upper body and lower extremities. Visual inspection and palpation techniques followed the guidelines established by the Academy of Nutrition and Dietetics and the American Society for Parenteral and Enteral Nutrition (14, 17–19), which ensured consistency in the evaluation and interpretation of findings for each body region.

For muscle mass assessment, the head and upper body were evaluated across the temple region (temporalis), clavicle and shoulder regions (pectoralis major, deltoid and trapezius), scapular region and upper back (trapezius, supraspinatus and infraspinatus) and hand regions, including the palmar (opponens pollicis, adductor pollicis and first dorsal interossei) and dorsal interosseous muscles. In the lower extremities, assessments were focused on the anterior thigh (quadriceps), patellar region (quadriceps) and posterior calf (gastrocnemius muscle). For participants unable to sit upright, assessments were performed in the supine position with knees flexed to allow access to the quadriceps and calf muscles while maintaining a neutral upper-body posture.

Participants with normal muscle status, as assessed through both palpation and visual inspection, presented with well-defined muscles in the temple area, nonprominent clavicles, rounded shoulders, no scapular depressions and well-rounded muscle bulges in the palmar, dorsal, thigh and calf regions. Those with mild to moderate malnutrition exhibited slight temple depressions, more prominent clavicle bones, mild protrusion of the acromion, moderate scapular and spinal depressions, and decreased muscle development in the palmar, dorsal, thigh and calf areas. By contrast, participants with severe malnutrition showed pronounced temple hollowing, protruding clavicles, squared shoulders, visible scapulae, significant spinal depressions, noticeable bone prominence in the dorsal and palmar regions and minimal muscle mass in the thighs and calves.

To evaluate fat mass, subcutaneous fat was assessed in the orbital region (around the eyes), the upper arm (triceps) and the thoracic and lumbar regions (mid-axillary line, ribs, iliac crest and lower back). The dietetics trainees assessed subcutaneous fat by palpating the orbital fat pads, pinching and rolling the triceps region with the arm flexed at 90° and palpating and inspecting the thoracic and lumbar regions while the participants stood with their backs pressed against a wall. In individuals with normal nutritional status, the findings included prominent fat pads around the eyes, adequate triceps fat and ribs that were not easily discernible. Participants with mild to moderate subcutaneous fat loss demonstrated slight darkening and a hollow appearance in the orbital region, limited triceps fat and ribs that were visible with minor depression. Severe subcutaneous fat loss was characterised by deep hollowing in the orbital region, minimal triceps fat and pronounced rib visibility with distinct depressions.

#### BIA

Before the BIA device was used, the participants’ medical histories were reviewed, and they were assessed for fluid imbalances, such as oedema or dehydration, as these conditions could affect the accuracy of the results. The SECA mBCA 525 BIA was used to evaluate skeletal muscle mass, fat-free mass index, fat mass and total body fat, in accordance with established protocols. The participants were instructed to relax and avoid consuming any food or beverages shortly before the measurements. During the assessment, they lay supine on the BIA machine to ensure proper positioning for accurate readings and optimal electrode contact. A low-level electrical current was then applied to the body to obtain measurements, and the data were recorded (20). The appendicular skeletal muscle index (ASMI) was calculated by dividing the total lean muscle mass of the upper and lower extremities by the square of each participant’s height (21). The fat mass index was determined by dividing the body fat mass (in kilograms) by the square of height (in metres) (22).

#### Sarcopenia

Sarcopenia is diagnosed based on the presence of a low ASMI combined with either reduced muscle strength or low or impaired physical performance (4). The threshold for a low ASMI, as measured by BIA, is < 7.0 kg/m^2^ for men and < 5.7 kg/m^2^ for women. Low muscle strength is defined as a handgrip strength of < 28 kg for men and < 18 kg for women.

### Statistical Analysis

Data were analysed using the Statistical Package for the Social Sciences (version 28.0, IBM, U.S.). Associations between NFPE-assessed body composition and i) BIA-derived body composition, ii) handgrip strength and iii) sarcopenia were analysed using McNemar’s test, with statistical significance set at a *P*-value < 0.05. Cohen’s kappa coefficient (κ) was calculated to examine the level of agreement between the variables. Kappa values were interpreted according to the criteria proposed by Landis and Koch (23).

## Results

### Participants’ Characteristics

A total of 74 patients with breast cancer consented to participate in this study. Table 1 presents the participants’ sociodemographic characteristics and medical data. The median age was 60.7 years (range between 50.5 and 67.3 years). Regarding monthly household income, most participants reported having no income; among those who did report income, approximately 29.7% were from low-income families, 17.6% from middle-income households and 6.8% from high-income households. The median duration since breast cancer diagnosis was 1.7 years (range between 0.7 and 5.3 years). Half of the participants were diagnosed with stage 2 cancer. Surgery was the primary treatment for 70.3% of the participants, and the majority had undergone at least two types of treatments.

### Participants’ Measurements

Table 2 provides an overview of the participants’ anthropometric data, handgrip strength, nutrition-focused physical findings, muscle and fat mass indices assessed using BIA and the prevalence of sarcopenia. Based on the WHO BMI classification, 55.4% of the participants were considered obese, 12.2% were overweight, and 4.1% were underweight. Based on handgrip strength measurements, most participants demonstrated normal muscle strength, while approximately one-third had reduced muscle strength.

NFPE muscle assessment revealed that 62.2% of the participants showed mild to moderate muscle wasting, while 37.8% were considered well nourished. In comparison, fat reserve evaluation showed that 60.8% of the participants were well nourished, and 39.2% had mild to moderate subcutaneous fat loss. BIA measurements indicated that nearly all the participants (97.2%) had low muscle mass, while only 2.8% had normal muscle mass. The majority of the participants (95.8%) demonstrated normal-to-high total body fat, whereas 4.2% had low total body fat. Based on reduced handgrip strength and a low ASMI, 26.8% of the participants were classified as sarcopenic according to the study’s diagnostic criteria.

### Association and Agreement Between NFPE-Assessed and BIA-Derived Muscle Status and Fat Status

Muscle and fat status evaluated using NFPE showed a significant association, respectively, with ASMI and total body fat derived by BIA (*P* < 0.001 for both) (Table 3). Agreement between NFPE and BIA was negligible for both muscle status (κ = 0.033; 95% confidence interval [CI]: −0.014, 0.080) and fat status (κ = 0.011; 95% CI: −0.065, 0.087). Similarly, NFPE-assessed muscle status showed no agreement with handgrip strength (*P* = 0.324) or sarcopenia status (*P* = 0.324) (Table 4). Cohen’s kappa analysis demonstrated no agreement between NFPE-assessed muscle status and handgrip strength (κ = −0.118; 95% CI: −0.332, 0.096) or sarcopenia status (κ = −0.190; 95% CI: −0.394, 0.014).

## Discussion

### Nutrition-Focused Physical Findings

In the NFPE-based assessment of muscle status, more than half of the patients with breast cancer (62.2%) were classified as having mild to moderate muscle wasting. This finding reflects the potential impact of treatment-related side effects on muscle mass loss, in addition to the effects of reduced dietary intake and increased nutrient requirements commonly seen during treatment. Neoadjuvant and adjuvant chemotherapies, commonly employed to slow tumour progression and improve disease-free survival in patients with breast cancer (24, 25), are known for their significant off-target effects. Preclinical and clinical studies have shown that chemotherapeutic agents contribute to skeletal muscle deconditioning, leading to increased fatigue in patients (26–31). Additionally, evidence from Wilson et al. (32) indicates that breast cancer, along with the chemotherapeutic agents used in its treatment, can interfere with primary protein synthesis pathways in skeletal muscle. This interference may disrupt protein turnover, highlighting the intricate relationship between cancer progression, therapeutic intervention and skeletal muscle metabolism.

### Association and Agreement Between NFPE-Assessed and BIA-Derived Muscle Status and Fat Status

Of the 71 participants evaluated, 43 (60.6%) classified as having mild to moderate muscle wasting by NFPE also demonstrated low muscle mass by BIA, with a significant association observed between the two measures (Table 3). NFPE and ASMI are both used in the assessment of muscle status but capture different dimensions of muscle health. NFPE offers a comprehensive evaluation of muscle status across body regions, whereas ASMI, derived from BIA, provides a quantitative measurement of skeletal muscle mass relative to height, making it particularly useful in the context of malnutrition and overall health assessment (33). These tools complement one another and contribute valuable insights into an individual’s nutritional and musculoskeletal health, thus facilitating more holistic healthcare management and treatment planning (34). NFPE can detect declines in muscle mass, loss of subcutaneous fat stores and clinical signs of malnutrition, such as skin alterations, oedema and poor wound healing, all of which can affect ASMI values. Additionally, ASMI has been shown to correlate with muscle strength metrics, such as handgrip strength, further supporting its relationship with overall muscle status (35, 36). The findings of this study underscore the significant association between NFPE-assessed muscle status and ASMI derived by BIA, offering valuable insights into muscle health, particularly when objective measures such as BIA are unavailable.

Regarding body fat, 56.3% of the participants who were categorised as well nourished by NFPE were found to have normal-to-high total body fat based on BIA, and this association was significant (Table 3). However, 28 participants (39.4%) who were measured as having normal-to-high total body fat by BIA were classified as having mild to moderate losses according to NFPE. This discrepancy could be attributed to the fact that NFPE focuses on specific regions of the body to assess fat loss rather than providing a comprehensive measurement of the total body fat percentage. NFPE predominantly evaluates fat loss at orbital, triceps, thoracic and lumbar regions. While this approach is effective for detecting fat depletion in localised regions, it does not offer precise quantification of the overall body fat composition (37). Methods such as DEXA and BIA provide accurate measurements of total body fat percentage. NFPE, being more qualitative and observational, is well suited for identifying signs of fat loss in specific body regions but may fall short in providing a comprehensive evaluation of total body fat status.

Although the agreement between NFPE and BIA was slight, the findings should be interpreted with caution because the kappa statistic is sensitive to the prevalence of the condition being measured (38, 39). In clinical populations where the distribution of muscle or fat is skewed, kappa may substantially underestimate the true degree of agreement between the two methods (40). A low kappa coefficient does not necessarily indicate that physical examination lacks validity or clinical utility (41). NFPE remains clinically relevant due to its practicality and bedside applicability. In addition, physical examination approaches for body composition assessment continue to evolve, with ongoing efforts to improve reproducibility and reliability in clinical practice.

### Association and Agreement of NFPE-Assessed Muscle Status with Handgrip Strength and Sarcopenia

This study found no association or agreement between overall muscle status as assessed by NFPE and handgrip strength (Table 4). Handgrip strength primarily reflects upper-body muscle function and may not accurately represent the total muscle mass or strength across the entire body. The differential susceptibility of muscle groups to malnutrition or disease further limits the ability of handgrip strength to reliably indicate overall muscle loss (42). By contrast, NFPE evaluates both the upper- and lower-body skeletal muscles, providing a comprehensive assessment of muscle status. Additionally, factors such as age, sex and hand dominance can influence handgrip strength, further complicating the interpretation of its results (42).

Similarly, there was no association or agreement between overall muscle status as evaluated by NFPE and the presence of sarcopenia (Table 4). Sarcopenia is characterised by loss of muscle mass and function, whereas NFPE primarily assesses structural indicators of malnutrition, including muscle wasting, subcutaneous fat loss and fluid accumulation, without directly evaluating functional parameters, such as muscle strength. Therefore, the diagnosis of sarcopenia requires a more comprehensive assessment that extends beyond the scope of NFPE.

### Strengths and Limitations of the Study

To the best of our knowledge, this is the first study in Malaysia to investigate the association and agreement between NFPE-assessed muscle status and i) BIA-derived ASMI; ii) handgrip strength; and iii) sarcopenia among patients with breast cancer. The significance of this study lies in its contribution to the limited body of research on muscle and fat assessment using a subjective data collection method. However, this study also has limitations. The accuracy and reliability of the data collected may have been influenced by the experience and technique of the dietetics trainees performing NFPE. Although skill levels may have varied, efforts were made to standardise training and ensure consistency in data collection procedures. Moreover, quality control measures were implemented to minimise discrepancies in NFPE interpretation.

## Conclusion

Muscle and fat status assessed by NFPE showed a statistically significant association with BIA-derived body composition measures, but agreement between the two methods was slight. NFPE offers a simple, cost-effective and readily accessible approach to body composition assessment, particularly in resource-limited hospital settings in which objective tools, such as BIA, may not be consistently available. As a bedside assessment requiring no specialised equipment, NFPE can be integrated into routine nutritional care without adding burden to clinical workflow. Dietitians should maintain competency in NFPE as part of comprehensive nutritional assessment, particularly when objective tools are inaccessible. In breast cancer patients, muscle wasting is a recognised concern related to both disease and treatment. Integrating body composition assessment into routine nutritional care may support more targeted, individualised nutrition interventions and improve clinical outcomes.

## Figures and Tables

**Table 1:** Participants’ sociodemographic and medical data (*N* = 74).

Variables	Frequency (%)	Median (IQR)
Age (years)		60.7 (50.5, 67.3)

Duration since breast cancer diagnosis (years)		1.7 (0.7, 5.3)

**Monthly household income**		

None	34 (45.9)	
< MYR4,850	22 (29.7)	
MYR4,851 to MYR10,959	13 (17.6)	
≥ MYR10,960	5 (6.8)	

**Cancer stage**		

Stage 1	18 (24.3)	
Stage 2	37 (50.0)	
Stage 3	19 (25.7)	

**Cancer-related treatment history**		

Chemotherapy		
Yes	46 (62.2)	
No	28 (37.8)	

Radiotherapy		
Yes	33 (44.6)	
No	41 (55.4)	

Surgery		
Yes	52 (70.3)	
No	22 (29.7)	

Hormonal therapy		
Yes	43 (58.1)	
No	31 (41.9)	

Number of treatment plans received		3.0 (2.0, 3.3)

**Comorbidities**		

Diabetes Mellitus		
Yes	14 (18.9)	
No	60 (81.1)	

Hypertension		
Yes	23 (31.1)	
No	51 (68.9)	

Dyslipidaemia		
Yes	21 (28.4)	
No	53 (71.6)	

Others		
Yes	18 (24.3)	
No	58 (75.7)	

**Table 2:** Participants’ measurements.

Variables	Frequency (%)	Mean ± SD	Median (IQR)
**Anthropometric measurements (** ***N*** ** = 74)**			

Weight (kg)			61.5 (52.6, 71.9)
Height (cm)		155.4 ± 5.3	

Body mass index (kg/m^2^)			25.1 (21.8, 29.8)
Underweight (< 18.5)	3 (4.1)		
Normal (18.5 to 24.9)	21 (28.4)		
Overweight (≥ 25.0)	9 (12.2)		
Obese (≥ 30.0)	41 (55.4)		

Handgrip strength (*N* = 74)		20.7 ± 4.6	
Low muscle strength^a^	21 (28.4)		
Normal muscle strength	53 (71.6)		

**Nutrition-focused physical findings ( ** ***N =*** ** 74)**			

Muscle status			
Mild to moderate wasting	46 (62.2)		
Well nourished	28 (37.8)		

Fat status			
Mild to moderate loss	29 (39.2)		
Well nourished	45 (60.8)		

**Bioelectrical impedance analysis (** ***N*** ** = 71)**			

Appendicular skeletal muscle index (ASMI) (kg/m^2^)		3.8 ± 0.9	
Low muscle mass^b^	69 (97.2)		
Normal muscle mass	2 (2.8)		

Fat mass index (kg/m^2^)			10.6 (8.0, 13.6)
Total body fat (%)		42.4 ± 9.2	
Low total body fat^c^	3 (4.2)		
Normal and high total body fat	68 (95.8)		

**Presence of sarcopenia (** ***N*** ** = 71)**			

Sarcopenic^d^	19 (26.8)		
Non-sarcopenic	52 (73.2)		

a Low muscle strength was defined as < 18 kg for women, based on the Asian Working Group for Sarcopenia (AWGS) cut-off values (4);

b Low muscle mass/ASMI was defined as < 5.7 kg/m^2^ for women, based on AWGS cut-off values (4);

c Low total body fat was defined according to reference values reported in the *American Journal of Clinical Nutrition* for people aged between 20 to 39 years (women: 21% to 32%), 40 to 59 years (women: 23% to 33%) and 60 to 79 years (women: 24% to 35%) (23);

d Sarcopenia was diagnosed according to AWGS diagnostic criteria using handgrip strength and ASMI data (4).

**Table 3:** Association of muscle and fat status assessed by NFPE and BIA.

Nutrition-focused physical findings	Number of patients			*P*-value	Unweighted kappa (95% CI)

Muscle status	Appendicular skeletal muscle index (ASMI)		Total		

	Low	Normal			
Mild to moderate wasting	43	2	45	< 0.001	0.033 (−0.014, 0.080)
Well nourished	26	0	26		

Total	69	2	71		

**Fat status**	**Total body fat**		**Total**		

	**Low**	**Normal-to-high**			

Mild to moderate loss	1	28	29	< 0.001	0.011 (−0.065, 0.087)
Well nourished	2	40	42		

Total	3	68	71		

*P*-values were determined using McNemar’s test for marginal homogeneity of two paired dichotomous variables.

**Table 4:** Association of muscle status assessed by NFPE with handgrip strength and presence of sarcopenia.

Nutrition-focused physical findings physical findings	Number of patients			*P*-value	Unweighted kappa (95% CI)

Muscle status	Handgrip strength		Total		

	Low muscle strength	Normal muscle strength			
Mild to moderate wasting	15	31	46	0.324	−0.118 (−0.332, 0.096)
Well nourished	6	22	28		

Total	21	53	74		

**Muscle status**	**Presence of sarcopenia**		**Total**		

	**Sarcopenic**	**Non-sarcopenic**			

Mild to moderate wasting	15	30	45	0.324	−0.190 (−0.394, 0.014)
Well nourished	4	22	26		

Total	19	52	71		

*P*-values were determined using McNemar’s test for marginal homogeneity of two paired dichotomous variables.
